# New Appliances and Things Medical

**Published:** 1903-07-25

**Authors:** 


### NEW APPLIANCES AND THINGS MEDICAL.

[We shall be glad to receive, at our Office, 28 & 29 Southampton Street Strand, London, W.C., from the manufacturers, specimens of all new preparations and appliances which may be brought out from time to time.]

### MOSELEY'S FOOD.

(MOSELEY'S FOOD, LIMITED, STOCKPORT.)

MOSELEY'S FOOD is a dextrinised cereal flour made by a special process; it has a pleasant flavour, and may be rapidly prepared without any additional cooking. It is intended both as an infant and invalid food; for both of these purposes it is admirably suited when prepared with milk and administered in proper quantities. We are glad to notice that the instructions for preparing the food for infants have been drawn up with due consideration of the physiological needs of the baby for whom it is intended. That is to say, a special table is provided in which the quantity for each meal, the total quantity for 24 hours, and the intervals between feedings are definitely stated: these figures naturally apply only to healthy infants; in special cases the quantities may have to be modified. Mothers or nurses who follow the instructions accurately will in no case go very far wrong, and in all probability will in the majority of cases meet with the greatest success.

